# Mental Illness Inequalities by Multimorbidity, Use of Health Resources and Socio-Economic Status in an Aging Society

**DOI:** 10.3390/ijerph18020458

**Published:** 2021-01-08

**Authors:** Manuel García-Goñi, Alexandrina P. Stoyanova, Roberto Nuño-Solinís

**Affiliations:** 1 Department of Applied & Structural Economics and History, Faculty of Economics and Business, Complutense University of Madrid, Campus de Somosaguas, 28223 Pozuelo de Alarcón (Madrid), Spain; 2 Department of Economics, Faculty of Economics and Business Administration, University of Barcelona, 08034 Barcelona, Spain; alexandrina.stoyanova@ub.edu; 3 Health Unit, Deusto Business School, University of Deusto, 48007 Bilbao, Spain; roberto.nuno@deusto.es

**Keywords:** mental illness, socioeconomic status, inequality, access to care, multimorbidity

## Abstract

Background: Mental illness, multi-morbidity, and socio-economic inequalities are some of the main challenges for the public health system nowadays, and are further aggravated by the process of population aging. Therefore, it is widely accepted that health systems need to focus their strategies for confronting such concerns. With guaranteed access to health care services under universal coverage in many health systems, it is expected that all services be provided equally to patients with the same level of need. Methods: In this paper, we explore the existence of inequalities in the access to services of patients with mental illness taking into account whether they are multimorbid patients, their socioeconomic status, and their age. We take advantage of a one-year (2010–2011) database on individual healthcare utilization and expenditures for the total population (N = 2,262,698) of the Basque Country. Results: More comorbidity leads to greater inequality in prevalence, being the poor sicker, although with age, this inequality decreases. All health services are more oriented towards greater utilization of the poor and sicker, particularly in the case of visits to specialists and emergency care. Conclusions: Mental health inequalities in prevalence have been identified as being disproportionally concentrated in the least affluent areas of the Basque Country. However, inequalities in the utilization of publicly-provided health services present a pro-poor orientation. As this region has adopted a system-wide transformation towards integrated care, its mental health delivery model offers excellent potential for international comparisons and benchlearning.

## 1. Introduction

Mental illness, multi-morbidity, and socio-economic inequalities are some of the main challenges for health systems nowadays and are further aggravated by the process of population aging. Therefore, it is widely accepted that health systems need to focus their strategies for confronting such concerns.

Mental disorders are among the major causes of ill-health and disability worldwide [1,2], accounting for an estimated 7% of all global disease as measured in disability-adjusted life-years [3], and have been found responsible for more of the global burden of disease than HIV/AIDS and tuberculosis, diabetes, or transport injuries [4]. Hence, the World Health Organization (WHO) considers that mental illness is a worldwide problem [5]. Mental ill-health also supposes an important economic burden, with estimated total costs of more than 4% of GDP, or over EUR 600 billion, across the 28 EU countries, from which, EUR 240 billion are due to lost productivity [6]. As a consequence, it is crucial for the health and social systems to tackle mental health problems. In that line, the World Health Organization has repeatedly recommended integrating mental health services into primary care settings (see, for instance, Funk and Ivbijaro, 2008 [7]). The impact of implementing mental health actions has also been analyzed, and as Layard (2016) [8] points out, mental health therapy boosts both employment and output, with gains exceeding the cost of treatment, producing savings in physical health care and improving life-satisfaction. The process of aging in the global population also has an impact on the incidence of mental disorders. In fact, Whiteford et al. [4] estimate that the burden of mental disorders together with substance use disorders increased by about 37% between 1990 and 2010. Besides, it is known that some specific mental conditions, like dementia or depression, are common in old age [9], while studies such as that of Barnett et al. (2012) [10] find that the relationship between the prevalence of mental health disorders and age increases until about the age of 60 years, and decreases afterward.

During the last decades, the change in the burden of disease evidenced by the increase in the prevalence of chronic diseases has become a major concern for health systems [11,12,13,14]. The change in the type of patients’ needs to be reflected in the way in which health services are provided. However, still, many health systems are organized to better provide an acute, reactive, and episodic model of care, instead of adapting to the preferences and needs of the growing number of patients with chronic health conditions [13]. Importantly, most of those chronic patients suffer from multiple chronic conditions and, thus, become multimorbid patients [15], making multimorbidity the most prevalent condition [16,17]. The likelihood of presenting multimorbidity increases with age [18,19] although it is a concern not only in the elderly population but also for the middle-aged population [10,20]. For that reason, the aging of the population is expected to emphasize the burden of disease toward chronic conditions. From the economic point of view, this challenge is even more important if we take into account the enormous concentration of health expenditures on chronic patients, which has been shown to account for 78% of total health expenditures in the US [17] or 70% in Spain [21], and the fact that the annual cost of healthcare for a given chronic patient increases with the number of coexisting conditions [19]. At the same time, older people suffer from an increased proportion of morbidity and mortality due to chronic disease, characterized by multimorbidity and complex health profiles [22]. Neurological and mental disorders are among the leading contributors to the disease burden in older people [23].

Universal health systems aim to provide equity in access to health insurance and health provision. Different notions of equity in access have been discussed traditionally in the literature (see, for instance, Oliver and Mossialos [24]), such as equal access to health care for those in equal need of care, or equal utilization of health care for those in equal need of care. The literature has found evidence that socioeconomic status (SES) is a good predictor of health, as it is also related to income, education, or occupation [25]. This relationship has also been found specifically for the elderly in China. In fact, lower SES is associated with greater access barriers to health care among the urban elderly [26], which derives in worse health status and premature death; and mortality and access to care would be reduced with more comprehensive health insurance that increased access to care [27].

Low socioeconomic status affects different aspects of social life, health status, and health care utilization among them. Dimensions of social inequalities in health are commonly measured by SES indexes or various indicators defined by individuals’ level of education, labor market status, income, or material wealth. Socioeconomic status is especially important for the elderly because they conform to a vulnerable group, being at higher risk of social and family isolation and loneliness, which may explain anxiety and depression in older age [28]. Feelings of isolation may arise due to retirement, which is associated with receiving a lower or a less stable income, or due to the loss of a partner or friends through illness. Vulnerability is also related to chronicity that may lead to deprivation of mobility, independence, and cognitive skills. At the same time, multimorbidity is related to SES, with a lower prevalence of multimorbidity for richer socioeconomic groups [29,30], and hence, the prevalence of mental health conditions is also correlated with the socioeconomic status. A social gradient in mental health status has been previously reported [31,32,33,34,35,36].

We focus our analysis on the prevalence of mental illness and study the existence of inequalities in multimorbidity and the use of health care resources by SES and age, which has an increasing relevance because of the aging process in our society. Although the literature, as presented, has extensively analyzed the impact of mental health on those variables separately, we contribute to the literature by focusing on the presence of mental illness but looking at the distribution of all other variables. If there are inequalities in the presence of mental disorders with other conditions due to socioeconomic status for the elderly, that will come with a greater relevance given their greater exposure to vulnerability and unfavorable socioeconomic circumstances.

Eckersley (2015) [37] explains how there is a common agreement in the existence of a relationship between inequality and health, and the convenience of further research about other social determinants of health. There are several theoretical approaches that aim to explain social inequalities in the prevalence of mental health problems. The social selection hypothesis [38] assumes that people with mental health problems descend the socioeconomic ladder due to their inability to meet expected role obligations because of their psychological condition. The social causation hypothesis [39] states that mental health problems arise from socioeconomic deprivation.

We take advantage of our analysis with a rich dataset containing clinical and administrative records from the Basque Country, a northern region in Spain. The Basque population, like the rest of the population in Spain, has universal health coverage, access to publicly-funded health care services is free at the point of delivery, with patients paying copayments only on prescribed medicines, the retired and some chronically ill being excluded [14,40]. We aim (i) to examine the distribution of four mental disorders in relation to age; (ii) to measure the socioeconomic inequality in mental health conditions for the entire population residing in the Basque Country, and; (iii) to determine whether an individual who suffers from a given mental disorder uses different types of health services depending on their SES. Finally, we discuss the implications of our findings for health policies in societies facing the problems of increasing chronicity and population aging.

## 2. Materials and Methods

### 2.1. Data

We utilized a database elaborated by the population stratification program (PREST) of the Basque Country, owned by the Basque Health Service and whose access is restricted. It included every individual covered on 31 August 2011 by the public health insurance in the Basque Country and who had been covered for at least 6 months in the previous year, regardless of whether they used the Basque Health Service or not. Hence, with universal health insurance, the dataset included practically the entire population of the Basque Country. This dataset had been used previously in the literature (see for instance [19] or [41]).

PREST dataset combines all clinical and administrative information available to the regional public health authorities, and thus, we had individual-level data on age, sex, health care use by type of services and diagnosis, small area of residence indicator, and socioeconomic status. The analyses presented here covered one year, from 1 September 2010 to 31 August 2011. The total sample included 2,262,698 individuals; slightly more than half of them were women (50.9%). The average age was 42.2 and 45.1 years for men and women, respectively. Children and adolescents (aged 16 years and below) represented 13.5% of the sample, while the elderly represented almost 20% of the population.

#### 2.1.1. Health Indicators

Following the national health policy guidelines set by the Ministry of Health, Social Services and Equality of Spain, Basque Health authorities codified diagnoses on hospital discharge forms, emergency department and primary care medical records according to ICD-9-CM [42], while the Anatomical Therapeutic Chemical (ATC) classification system [43] is used for drugs prescribed by general practitioners. With this information, each Basque resident is assigned to Adjusted Clinical Groups (ACGs), a case-mix system that allows the detection of health problems from diagnoses and prescriptions and expected future health care needs and related costs [44].

Our choice of mental health indicators was based on relevance, as measured by prevalence in the study population greater than 0.5%, and was consistent with different studies in Spanish [45,46] and international [30] literature. Thus, we proxied mental health by four chronic conditions: (i) anxiety, and other neurotic, stress-related and somatoform disorders (10.7% of the sample); (ii) depression (3.5%); (iii) dementia (1.7%), and; (iv) schizophrenia, affective psychosis or bipolar disorder (0.7%). Other conditions considered in the sample had a lower prevalence: alcohol (0.39%), substance abuse (0.21%), anorexia and bulimia (0.15%), or ADHD (0.07%).

Multimorbidity was defined as the coexistence of two or more chronic conditions in the same patient. As we focused our attention on patients with mental health conditions, multimorbidity was defined as the presence of at least one additional chronic disease to the existing mental health condition. We followed the methodology proposed by Barnett et al. (2012) [10] and Orueta et al. (2014) [19] to use a list of the 52 more relevant chronic conditions for our population, including asthma, chronic kidney disease, diabetes, chronic obstructive pulmonary disease, heart failure, HIV, migraine, or multiple sclerosis. We considered multimorbidity here for two reasons. Firstly, multimorbidity is prevalent and increases with age [10,44]. And, secondly, the prevalence of physical and mental health morbidity is significantly higher among the elderly [10], it causes the greatest decrements in quality of life [47,48,49], and is also associated with poorer outcomes in treating physical chronic conditions, increased mortality [50], and more intensive use of health services [51,52].

#### 2.1.2. Socioeconomic Status (SES)

Individual SES is based on the deprivation index (DI) elaborated by the MEDEA project in 2008 [53]. The DI categorizes individuals into five socioeconomic status groups (quintiles) according to their area of residence. The DI is calculated by taking into account five dimensions: the percentages of residents in the area who are manual workers, the percentage of unemployed, of temporary employees, and the percentage of employees with an inadequate level of educational attainment, overall, and specifically among the young population between 16 and 29 years (Domínguez-Berjón et al. 2008) [53]. Area-level deprivation measures have been commonly used in the literature of health inequalities, as reviewed by Ingram et al. (2020) [54]. The main finding is that those living in the most deprived areas had the highest prevalence or incidence of multimorbidity.

#### 2.1.3. Health Care

Health care is measured based on the cost-weighted actual use of health care services over a year. Our dataset contained information on the following types of services: primary care (that included visits to general practitioners, nurses, laboratory tests, and radiology examinations), specialist care (visits to specialists, rehabilitation, dialysis, radiotherapy, and chemotherapy), emergency care, use of pharmaceuticals (extracted from primary care prescriptions recorded in electronic health records), and inpatient stays in general hospitals. Hospitalizations in monographic psychiatric hospitals were not included in our analysis as they were not available in the dataset. We used all the contacts within the health system, coded, as mentioned before, according to ICD-9-CM [42], and all expenditure in prescribed pharmaceuticals coded by the ATC classification system [44] to assign costs to the different diagnoses. This method had been previously applied to the same dataset by Orueta et al. [19].

The primary and specialized care expenditures were calculated by multiplying the number of services used by each individual by a standardized cost, calculated as the average cost per visit or use of those health services among their total cost. The cost of inpatient care in general hospitals was determined according to its weight in the corresponding Diagnosis-Related Groups (DRGs). Finally, the cost of prescribed pharmaceuticals was based on market prices following the registry of pharmaceutical expenditures through prescriptions in the Basque Health System during the length of our study. We estimated a linear regression to assign the cost associated to each individual because of the provision of health services from several providers to all the different diagnoses registered by each patient following the conventional risk adjustment methodology (see, for instance, Van de Ven and Ellis, 2000 [55]).

### 2.2. Methods

To assess socioeconomic inequality in mental health in this study, we calculated the concentration index (*CI*) [56]. The *CI* allowed measuring the distribution of one variable of interest (mental health status in our study) against the ranking of another, usually socioeconomic variables (here, the deprivation index of the area of residence), and ranges were between −1 and +1. When the variable of interest was the presence of a mental chronic condition, negative (positive) values of the *CI* indicated that there was socioeconomic inequality in favor of the worse-off (better-off). If the *CI* equaled 0, there was no evidence of inequality in mental health that could be attributed to socioeconomic status.

There are different ways of formally expressing the *CI*. The one that was most convenient for our analysis was the one proposed by Wagstaff (2002) [57] and it can be written as follows:

(1) $$CI=1-\left(2*\left(1-R_{i}\right)\right)\overset{n}{\underset{i=1}{\sum }}\frac{h_{i}}{n\mu }$$

where *n* is the sample size; *h_i_* is the mental health indicator; *μ* is the mean of mental health in the population; and *R_i_* is the relative rank in the socioeconomic distribution of the *i*th individual, being the 1st person, the poorest one (*i* = 1) and the *n*th person, the wealthiest one (*i = n*).

Alternatively, the concentration index could be calculated using the ‘convenient regression’ expression based on the covariance between $h_{i}$ and the ith individual relative rank, $R_{i}$:

(2) $$CI=\frac{2}{\mu }cov\left(h_{i},R_{i}\right)$$

The *CI* is a widely used measure of socioeconomic inequality in health and health care because it satisfies a number of desirable properties [58]. Its major advantage is the fact that it summarises the level of inequality in a single indicator that is easily comparable across regions (countries, areas of residence, etc.), time, use of different types of services, or age or disease groups [59]. Nevertheless, it also presented some important problems. In the case of binary indicators, like the ones we used in this study, the *CI* may depend on the mean of the health variable; not allowing for comparisons across population groups with different mean heath levels [60]. In order to deal with the binary nature of our mental health indicators, we followed the correction mechanism proposed by Wagstaff (2005) [60]. Thus, we were able to compare the extent of socioeconomic inequality for each of the mental health chronic conditions (with varying levels of prevalence). We calculated the *CI* separately for men and women and for all age groups, to be able to compare the status of the elderly with respect to the population.

Concentration indices for actual health care use reflected inequalities in the amount of care different individuals got. However, in order to assess the degree of inequity in the use of health care, we needed to adjust for the varying needs among individuals. Therefore, we calculated the horizontal inequity index (*HI*) as the difference between the actual health care utilization inequality (observed *CI*) and the estimated inequality associated to need [61]. Need-predicted health care use was derived from the predictions of an ordinary least-squares regression model of health care against a number of need (age, gender, and the number of morbidities in our study) and non-need variables (the deprivation index of the area of residence). Thus, we obtained the level of care each individual would have received had he/she been treated in the same way as were, on average, other individuals with equal needs. To avoid biased estimates of the coefficients of the need-related indicators, all non-need variables were set to their mean, thus neutralizing their effect [62]. In our analysis, we used the statistical significance criterion in order to be comparable with other papers in the literature [63,64,65,66].

### 2.3. Ethics Statement

The Clinical Research Ethics Committee of the Basque Country approved this study according to the Spanish Law 14/2007 on Biomedical Research, the Ethical Principles for Medical Research of the Declaration of Helsinki, and other applicable ethical principles. We used databases that employed an opaque identifier to ensure patient confidentiality. Written consent by the patients was specifically waived by the approving Committee.

## 3. Results

### 3.1. Descriptive Statistics

**Figure 1:**
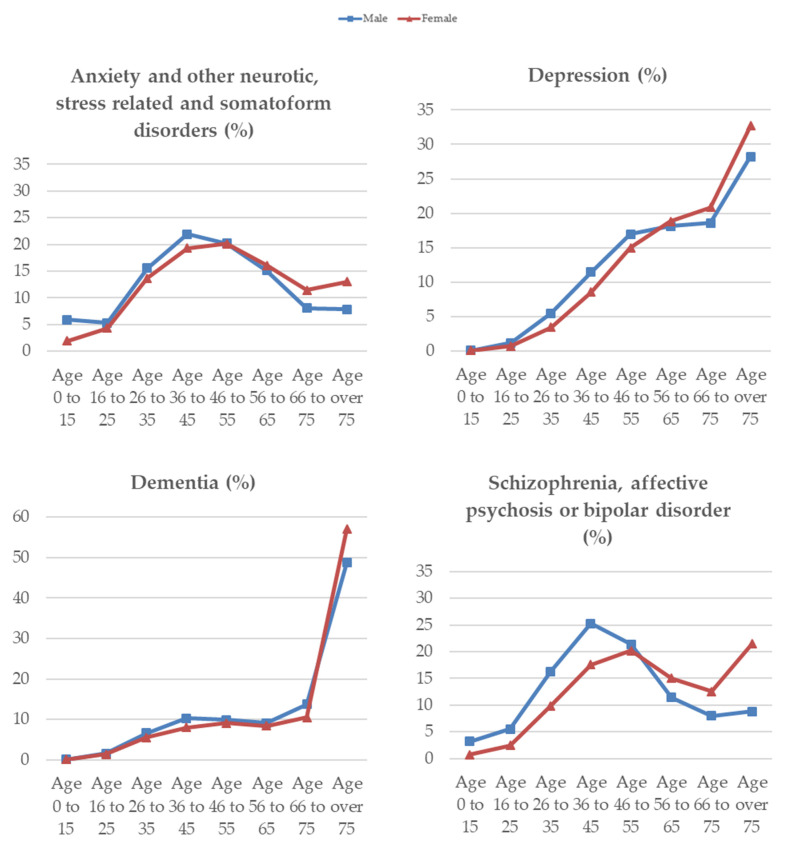
Distribution of mental health disorders by age and gender in the Basque Country.

The percentage of the healthy population in the Basque Country (not suffering from physical or mental conditions) was 57.08%. Table 1 summarizes the information regarding the general Basque population and the prevalence of mental health disorders by age groups, gender, and socioeconomic status (based on the deprivation index of the area of residence) contained in the PREST dataset. One out of ten Basque residents was diagnosed with anxiety or other neurotic, stress-related, and somatoform disorders, the prevalence was 107.277 per 1000 individuals. Women were, in general, much more prone to suffer from these disorders than men (prevalence rate of 140.651 for women and 72.683 for men). However, an interesting pattern emerged when we looked at the distribution of people with anxiety and other neurotic, stress-related, and somatoform disorders by age (see Figure 1). The prevalence by age was an inverted-U shaped, being the highest among the young middle-aged men (36 to 45 years of age) and the women aged 46 to 55 years. Socioeconomic status acted as a protector. Prevalence of anxiety and related disorders decreased with socioeconomic status, more for women than for men.

Depression hit 35.284 individuals out of every 1000 and the prevalence increased with age. The prevalence of depression was nearly three times higher in women than in men (59.894 versus 19.994, respectively). Interestingly, deprivation was positively related to depressive disorders only for women, while the rates of prevalence only slightly varied by socioeconomic status for men.

**Figure 2:**
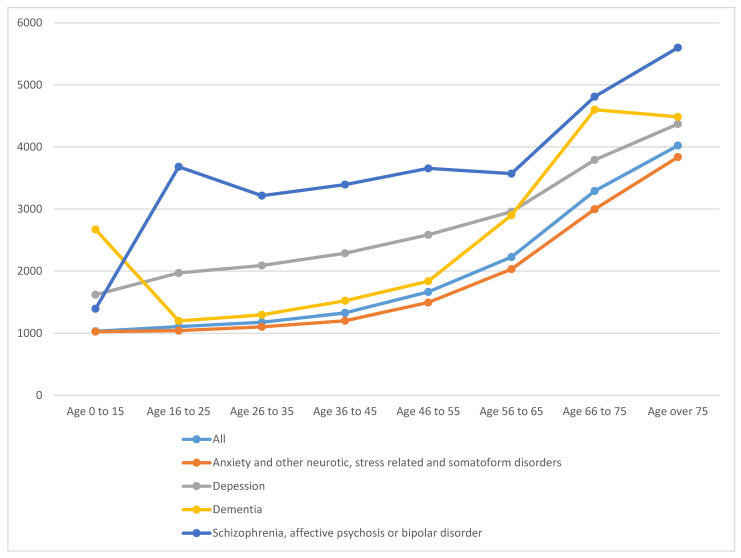
Total health expenditures by mental health disorder and age groups.

Average total health expenditures and costs by type of health services, calculated on the basis of actual health services utilization, by mental health disorder and age groups are shown in Table 2 and Figure 2. Average health care expenditures amounted to 1133€ for any Basque individual, with inpatients in general hospitals representing one-third of these. Health care costs for those suffering from mental disorders were considerably higher and the distribution of the costs among the different types of services differed for these individuals. The costs of health care provided to individuals diagnosed with anxiety and other neurotic, stress-related, and somatoform disorders increased to 1847€ and although the functional distribution of these costs was similar to the one of the general population, the weight of specialized care was slightly higher in detriment to hospital services. Health care expenditures for people with depression, dementia, or severe mental health disorders were at least three times higher than the average. Inpatient stays in general hospitals were the most expensive type of care used by those diagnosed with depressive symptoms, representing 32% of total costs, while for patients with dementia, hospital care represented almost half of total health care expenditures. For those diagnosed with severe mental health disorders, hospital and specialized (932€) care accounted for two-thirds of all health expenditures.

### 3.2. Inequality in Mental Health

Table 3 represents the main results regarding socioeconomic inequalities in mental health in the Basque country. The overall concentration indices with age and sex adjusted were negative, meaning that the burden of mental health disorders was disproportionately concentrated among the individuals residing in more deprived areas. Although this general finding held for both men and women, the degree of the socioeconomic gradient in the prevalence of the different mental health conditions varied by gender. The inequalities in common mental health disorders (anxiety and depressive disorders) were most pronounced among females. The opposite was observed for severe mental health conditions and dementia, where the extent of inequality was larger for men, but the gender gap was not that big. With respect to age, we observed that the burden of mental health disorders spanned all age groups and there was evidence of statistically significant socioeconomic inequalities (with a few exceptions). The distribution of these inequalities was pro-rich, with individuals residing in less affluent areas concentrating the highest rates of prevalence of mental health problems. Interestingly, the magnitude of the socioeconomic inequalities in common mental disorders and dementia decreased with age. In the case of dementia, in fact, inequalities not only steadily decreased with age, but dementias seem to be disproportionately concentrated among the rich for the elderly.

The results regarding socioeconomic inequality in multimorbidity by mental health problem are shown in Table 4. The overall negative concentration indices revealed that, after controlling for age and sex, people residing in more deprived areas presented more chronic conditions compared to those who lived in more affluent areas. The only positive index was the one corresponding to no chronic conditions at all, meaning that better health was more concentrated among the rich. As expected, and previously observed [59], the magnitude of socioeconomic inequality in multimorbidity increased with the number of chronic conditions. However, a different picture emerged when we took a closer look at the inequalities in multimorbidity among those diagnosed with mental health disorders. There was pro-rich inequality in multimorbidity among those individuals who suffered from any of the four problems studied here who also had three or fewer additional chronic conditions (not all of them statistically significant). From then, inequalities were concentrated among the most disadvantaged. Having nine or more chronic conditions on top of mental health disorders was significantly disproportionately concentrated among the poorer. The magnitude of the indices was notably higher than for the lower levels of multimorbidity.

### 3.3. Inequity in Health Care Use among Individuals with Mental Health Disorders

For all types of health care and for all mental health disorders, the concentration indices of observed costs were negative and statistically significant (except for the *CI* of pharmaceutical expenditures among those who suffered from dementia), which indicated that individuals living in less affluent areas were more likely to use these services (Table 5). The socioeconomic gradient was markedly higher for the use of emergency care, specialized services, and hospitalizations compared to the degree of inequality for pharmaceuticals and primary care for all four mental health conditions. That differed from the total sample estimates of inequality, where costs of medicines and other pharmaceutical products were much more concentrated among those residing in poorer areas, while emergency care was the least unequally distributed by SES.

## 4. Discussion

Public health authorities are responsible for the use of resources within the National Health System and one of their goals is to guarantee equal access to health services based on need, which can be inferred by prevalence.

Prevalence of mental illness depends on age and gender but also on socioeconomic status, and the health status of patients classified by the number of chronic conditions they suffer. Our result was consistent with the international literature focused on multimorbidity, such as [10,29,30,53] or [54] showing that lower SES, age, and gender are associated with multimorbidity, although, for instance, in Schiøtz et al. (2017) [30], SES is given differently by educational attainment. In our study, focused on inequalities in mental health, we find that multimorbidity leads to greater inequality in the prevalence of mental illnesses, being the poorer the sicker, although with age, this inequality decreases.

We measure inequality in access to care through the utilization of public health services leading us to individual health expenditures derived from the provision of these services, controlling for all the relevant variables. Health care costs for those suffering from mental disorders are considerably higher and the distribution of the costs among the different types of services differs for these individuals, being inpatient stays, pharmaceutical prescriptions and specialist care the providers presenting the highest cost. We find greater utilization of the poor and sicker particularly in the case of specialized and emergency care. With respect to age, individual health expenditures are higher for older patients with mental disorders, which is consistent with them suffering from more chronic conditions.

The pro-poor inequality in the use of each type of care seems to reflect, in large part, the unequal distribution of need. After controlling for need, inequalities in the use of care by socioeconomic status are much attenuated. The indices for horizontal inequity are still negative, but lower in magnitude and even become non-significant for some types of care and mental health conditions. Overall, poorer individuals use significantly more than their need-predicted share of health services, especially for specialized care and emergency care. The use of pharmaceuticals is only slightly unequally distributed among the poor with anxiety and other stress-related disorders, among the rich who suffer from dementia, and does not show inequity for those diagnosed with depression or severe mental disorders. Hence, the use is biased towards those in greater need, and it would seem that access to health services within the public system is not a major problem for mental illness patients in the Basque Country. This finding is consistent with previous studies on this topic performed in this region [59] but this pro-poor orientation has not been found in other studies in Spain [67,68]. 

However, when taking into account simultaneously multimorbidity, socioeconomic status, and the presence of mental illness, we have identified that there is pro-rich inequality for those with less than three additional chronic conditions, and pro-poor in those suffering from four or more additional chronic conditions. That indicates, that the multimorbidity condition (positively correlated for the elderly) with mental illnesses is much more severe in poor than in rich neighborhoods. As in Schiøtz et al. (2017) [30], we interpret that low SES may be correlated with poor childhood conditions, working environments, and lifestyles that might lead to less healthy behaviors such as smoking or diet increasing differences in the prevalence of multimorbidity [30,69] in the aged population and in particular for those with mental health conditions.

Even in countries where access to health services is guaranteed and does not seem to be a major concern, the diversity of health policies at a regional level can explain these inequalities on the pro-poor or pro-rich orientation of the publicly–funded mental healthcare provision. For instance, the Basque Country Health System has been a pioneer in the adoption of chronic care strategies and the emerging healthcare delivery model offers excellent potential for international comparisons, given its specific characteristics: a relatively homogeneous system providing universal coverage; a model based on primary care in the community; good integration of primary care and mental health; and successful implementation of a model of comprehensive care for chronic conditions [70,71]. At the same time, the health system should promote the right continuity and coordination of care for individuals suffering from mental health conditions and other chronic conditions [10,72]. Additionally, inequalities are being alleviated through the co-creation of value in the design of public provision of both health and social services addressed to those in need, as it has been shown that social disconnectedness could affect perceived isolation and symptoms of depression and anxiety among the elderly [73,74]. In the absence of well-designed access to social services and social care, an increase in socioeconomic inequalities can be expected, especially for the elderly, as a more vulnerable population. Hence, health care planning and organization should consider the socioeconomic gradient taking into account a greater presence of multimorbidity in aged and vulnerable individuals suffering from mental conditions.

Anyway, this final element is a limitation of our study as the available dataset does not contain information regarding social services for patients with mental health problems. Two other limitations related to the dataset should be noted, the lack of data on hospitalizations on monographic psychiatric hospitals and the absence of information on privately-funded healthcare utilization. Regarding the psychiatric hospitalizations not included in our dataset, they are related to patients with serious mental illness and publicly-funded, as privately-funded services for this severe profile of patients are almost non-existent in the studied region.

## 5. Conclusions

Mental illness, multimorbidity, and socioeconomic inequalities represent important challenges for the public health system in many countries, even more so due to the aging of the population. In this paper, we examine the prevalence of mental illnesses and the use of public health services by their patients, taking into account whether they suffer multiple chronic conditions, their socioeconomic status, and their age. We take advantage of data from the regional Basque Health System for practically the entire population (over two million) of the Basque Country.

We find inequalities in the prevalence of mental illness, which is significantly greater in more deprived economic areas, although with age, this inequality decreases. Even more, when taking into account the existence of other chronic conditions in mental health patients, it is in more deprived areas where we find more multimorbid patients with mental illness conditions. We also find some inequalities in the use of resources, in all types of health services, they are more oriented towards greater utilization of the poor and sicker, particularly in the case of visits to specialists and emergency care. Belonging to the National Health System in Spain, our results are consistent with the guaranteed equity in access to public health services. But, even if access is not a concern, socioeconomic inequalities in the prevalence remain that are space- and time-dependent and are multiple rather than singular. Managers and decision-makers should not take them as ‘given’ but as something that can be tackled with policies and well-targeted interventions, resulting in a pro-poor orientation like the case of the Basque Country has shown. This Region has adopted a system-wide transformation towards integrated care [74], oriented to be based on primary care in the community; good integration of primary care and mental health; and successful implementation of a model of comprehensive care for chronic conditions, promoting the right continuity and coordination of care for individuals suffering mental health conditions and other chronic conditions. The emerging mental health delivery model offers excellent potential for international comparisons and benchlearning.

## Figures and Tables

**Table 1:** Prevalence of mental health disorders by age groups, gender, and socioeconomic status in the Basque Country.

	Target Population		Anxiety and Other Neurotic, Stress-Related and Somatoform Disorders		Prevalence Ratio per 1000 Population	Depression		Prevalence Ratio per 1000 Population	Dementia		Prevalence Ratio per 1000 Population	Schizophrenia, Affective Psychosis or Bipolar Disorder		Prevalence Ratio per 1000 Population
	*n*	%	*n*	%		*n*	%		*n*	%		*n*	%	
All	2,262,698	100	242,736	100	107.277	79,838	100	35.284	37,449	100	16.551	15,141	100	6.692
Sex														
Males	1,111,050	49.10	80,755	33.27	72.683	19,944	24.98	17.951	12,308	32.87	11.078	8068	53.29	7.262
Females	1,151,648	50.90	161,981	66.73	140.651	59,894	75.02	52.007	25,141	67.13	21.830	7073	46.71	6.142
Age groups														
Age 0 to 15	305,753		7999			25			38			311		
Males	157,884	51.64	4798	59.98	30.389	11	44.00	0.070	16	42.11	0.101	257	82.64	1.628
Females	147,689	48.30	3201	40.02	21.674	14	56.00	0.095	22	57.89	0.149	54	17.36	0.366
Age 16 to 25	192,636		11,361			625			543			619		
Males	98,802	51.29	4304	37.88	43.562	231	36.96	2.338	191	35.17	1.933	447	72.21	4.524
Females	93,834	48.71	7057	62.12	75.207	394	63.04	4.199	352	64.83	3.751	172	27.79	1.833
Age 26 to 35	331,211		34,734			3136			2200			2016		
Males	169,592	51.20	12,543	36.11	73.960	1092	34.82	6.439	816	37.09	4.812	1316	65.28	7.760
Females	161,619	48.80	22,191	63.89	137.304	2044	65.18	12.647	1384	62.91	8.563	700	34.72	4.331
Age 36 to 45	384,784		48,927			7377			3288			3287		
Males	197,378	51.30	17,690	36.16	89.625	2277	30.87	11.536	1270	38.63	6.434	2041	62.09	10.341
Females	187,406	48.70	31,237	63.84	166.681	5100	69.13	27.214	2018	61.37	10.768	1246	37.91	6.649
Age 46 to 55	345,705		48,982			12,354			3495			3155		
Males	171,859	49.71	16,328	33.33	95.008	3384	27.39	19.691	1213	34.71	7.058	1727	54.74	10.049
Females	173,855	50.29	32,654	66.67	187.823	8970	72.61	51.595	2282	65.29	13.126	1428	45.26	8.214
Age 56 to 65	275,701		38,196			14,892			3230			1986		
Males	135,372	49.10	12,183	31.90	89.996	3613	24.26	26.689	1120	34.67	8.273	922	46.42	6.811
Females	140,329	50.90	26,013	68.10	185.372	11,279	75.74	80.375	2110	65.33	15.036	1064	53.58	7.582
Age 66 to 75	204,458		25,129			16,148			4332			1537		
Males	95,303	46.61	6548	26.06	68.707	3709	22.97	38.918	1688	38.97	17.712	646	42.03	6.778
Females	109,155	53.39	18,581	73.94	170.226	12,439	77.03	113.957	2644	61.03	24.222	891	57.97	8.163
Age over 75	222,630		27,408			25,227			20,323			2230		
Males	84,869	41.51	6361	25.31	74.951	5627	22.31	66.302	5994	29.49	70.626	712	31.93	8.389
Females	137,761	67.38	21,047	83.76	152.779	19,600	77.69	142.275	14,329	70.51	104.013	1518	68.07	11.019
Socioeconomic Status (quintiles)														
SES1 (highest)	479,316		43,774			15,482			7491			2851		
Males	228,747	47.72	14,066	32.13	61.492	3976	25.68	17.382	2379	31.76	10.400	1449	50.82	6.335
Females	250,569	52.28	29,708	67.87	118.562	11,506	74.32	45.919	5112	68.24	20.402	1402	49.18	5.595
SES2	487,140		50,072			16,422			7534			3034		
Males	238,951	49.05	16,602	33.16	69.479	4168	25.38	17.443	2413	32.03	10.098	1646	54.25	6.888
Females	248,189	50.95	33,470	66.84	134.857	12,254	74.62	49.374	5121	67.97	20.633	1388	45.75	5.593
SES3	457,665		50,145			16,245			7269			3088		
Males	226,345	49.46	16,774	33.45	74.108	4054	24.96	17.911	2454	33.76	10.842	1656	53.63	7.316
Females	231,320	50.54	33,371	66.55	144.263	12,191	75.04	52.702	4815	66.24	20.815	1432	46.37	6.191
SES4	422,729		47,917			15,694			7484			3030		
Males	209,966	49.67	16,187	33.78	77.093	3952	25.18	18.822	2553	34.11	12.159	1620	53.47	7.716
Females	212,763	50.33	31,730	66.22	149.133	11,742	74.82	55.188	4931	65.89	23.176	1410	46.53	6.627
SES5 (lowest)	415,848		50,828			15,995			7671			3138		
Males	207,041	49.79	17,126	33.69	82.718	3794	23.72	18.325	2509	32.71	12.118	1697	54.08	8.196
Females	208,807	50.21	33,702	66.31	161.403	12,201	76.28	58.432	5162	67.29	24.721	1441	45.92	6.901

**Table 2:** Total health care costs and costs (per year) for different types of health care services for individuals with and without mental disorders.

Health Care Services	All		Anxiety and Other Neurotic, Stress-Related and Somatoform Disorders		Depression		Dementia		Schizophrenia, Affective Psychosis or Bipolar Disorder		No Mental Health Disorders *	
	Mean (€) (Std.Dev.)	%	Mean (€) (Std.Dev.)	%	Mean (€) (Std.Dev.)	%	Mean (€) (Std.Dev.)	%	Mean (€) (Std.Dev.)	%	Mean (€) (Std.Dev.)	%
Primary care	261.14 (327.62)	23.05	424.19 (385.95)	22.97	636.67 (465.55)	18.67	566.88 (548.94)	15.67	460.06 (473.32)	11.84	229.14 (298.31)	24.25
Specialist care	257.08 (999.97)	22.69	454.41 (1168.73)	24.60	639.36 (1488.21)	18.74	491.49 (1410.76)	13.59	932.16 (1676.79)	23.98	219.04 (946.01)	23.19
Emergency care	54.20 (142.78)	4.78	85.48 (208.99)	4.63	94.87 (217.99)	2.78	114.78 (244.75)	3.17	135.52 (319.32)	3.49	48.90 (129.38)	5.17
Inpatient stays in general hospitals	376.29 (2294.01)	33.21	588.01 (2804.42)	31.84	1092.68 (3874.26)	32.03	1646.42 (4898.19)	45.52	1664.43 (4685.37)	42.82	314.36 (2090.98)	33.27
Prescription drugs and pharmaceutical products	175.21 (513.43)	15.47	294.74 (644.16)	15.96	947.40 (1046.47)	27.78	797.90 (1055.04)	22.06	694.65 (1225.34)	17.87	133.50 (433.35)	14.17
Total Health Care costs	1132.90 (3152.11)	100	1846.84 (3814.89)	100.00	3410.98 (5140.61)	100.00	3617.47 (6022.26)	100.00	3886.82 (5867.48)	100.00	944.94 (2846.28)	100.00

Note: * Individuals who do not present any of the following four mental health conditions: (i) anxiety and other neurotic, stress related and somatoform disorders; (ii) depression; (iii) dementia and, (iv) schizophrenia, affective psychosis or bipolar disorder.

**Table 3:** Socioeconomic inequality in mental health disorders by gender and age groups.

	Anxiety and Other Neurotic, Stress-Related and Somatoform Disorders		Depression		Dementia		Schizophrenia, Affective Psychosis or Bipolar Disorder	
	*CI*	Std.Dev.	*CI*	Std.Dev.	*CI*	Std.Dev.	*CI*	Std.Dev.
Total ^(a)^	−0.067 *	−54.654	−0.041 *	−20.074	−0.030 *	−10.068	−0.051 *	−10.891
Gender ^(b)^								
Female	−0.071 *	−46.072	−0.050 *	−21.119	−0.029 *	8.275	−0.049 *	−7.153
Male	−0.064 *	−30.412	−0.015 *	−3.745	−0.031 *	−6.000	−0.053 *	−8.130
Age groups								
0 to 15	−0.165 *	−24.293	−0.241 **	−1.928	−0.298 *	−2.830	−0.111 *	−3.327
16 to 25	−0.130 *	−22.851	−0.096 *	−4.110	−0.157 *	−6.115	−0.033	−1.419
26 to 35	−0.104 *	−31.483	−0.051 *	−4.876	−0.114 *	−9.043	−0.051 *	−3.961
36 to 45	−0.076 *	−27.213	−0.071 *	−10.365	−0.110 *	−10.685	−0.060 *	−5.950
46 to 55	−0.048 *	−17.169	−0.058 *	−10.918	−0.080 *	−8.112	−0.078 *	−7.442
56 to 65	−0.044 *	−13.985	−0.068 *	−13.905	−0.071 *	−6.914	−0.078 *	−5.939
66 to 75	−0.050 *	−12.982	−0.037 *	−7.941	−0.044 *	−5.068	−0.003	−0.208
Over 75	−0.049 *	−13.163	−0.013 *	−3.322	0.011 *	2.551	−0.011	−0.905

Note: * *p* < 0.05; ** *p* < 0.1. ^(a)^ Age and sex-adjusted *CI*. ^(b)^ Age-adjusted *CI*. All *CI*s are adjusted for clustering at the level if the general practice where the individual is registered.

**Table 4:** Socioeconomic inequality in multimorbidity among those with mental health disorders in the Basque Country.

	All		Anxiety and Other Neurotic, Stress-Related and Somatoform Disorders		Depression		Dementia		Schizophrenia, Affective Psychosis or BipoLar Disorder	
	*CI*	Std. Err.	*CI*	Std. Err.	*CI*	Std. Err.	*CI*	Std. Err.	*CI*	Std. Err.
0 conditions	0.067 *	101.342								
1 conditions	−0.030 *	−31.373	0.045 *	21.617	0.091 *	11.837	0.047 *	5.964	0.044 *	6.026
2 conditions	−0.040 *	−31.901	0.001	0.498	0.055 *	11.160	0.035 *	4.719	0.006	0.542
3 conditions	−0.048 *	−30.600	−0.012 *	−3.511	0.015 *	3.085	0.018 *	2.291	−0.008	−0.636
4 conditions	−0.062 *	−30.119	−0.032 *	−8.053	−0.011 **	−2.097	0.003	0.352	−0.015	−1.081
5 conditions	−0.069 *	−25.944	−0.045 *	−9.203	−0.036 *	−6.117	−0.027 *	−2.975	−0.036 **	−1.961
6 conditions	−0.075 *	−20.522	−0.047 *	−7.264	−0.047 *	−6.583	−0.041 *	−3.828	−0.046 **	−2.023
7 conditions	−0.085 *	−17.113	−0.058 *	−6.894	−0.035 *	−3.859	−0.041 *	−3.170	−0.056 **	−2.029
8 conditions	−0.084 *	−12.494	−0.049 *	−4.395	−0.056 *	−4.883	−0.029 **	1.824	0.001	0.020
9 conditions	−0.116 *	−12.468	−0.114 *	−7.551	−0.100 *	−6.596	−0.076 *	−3.898	−0.084 *	−2.165
10 or more conditions	−0.121 *	−14.129	−0.112 *	−7.939	−0.122 *	−8.504	−0.112 *	−6.301	−0.177 *	−5.175

Note: * *p* < 0.05; ** *p* < 0.1. Age and sex-adjusted *CI*. All *CI*s are adjusted for clustering at the level if the general practice where the individual is registered.

**Table 5:** Socioeconomic inequity in health care utilization (measured by costs) in the Basque Country.

	All		Anxiety and Other Neurotic, Stress-Related and Somatoform Disorders		Depression		Dementia		Schizophrenia, Affective Psychosis or Bipolar Disorder	
	Observed *CI* ^(a)^	*HI* ^(b)^	Observed *CI*	*HI*	Observed *CI*	*HI*	Observed *CI*	*HI*	Observed *CI*	*HI*
Primary care	−0.064 *	−0.035 *	−0.035 *	−0.017 *	−0.036 *	−0.021 *	−0.022 *	−0.011 *	−0.035 *	−0.016 *
Specialist care	−0.093 *	−0.047 *	−0.052 *	−0.032 *	−0.068 *	−0.046 *	−0.056 *	−0.032 *	−0.048 *	−0.034 *
Inpatient stays in general hospitals	−0.097 *	−0.020	−0.056 *	−0.006 *	−0.057 *	−0.013 *	−0.034 *	−0.009	−0.046 *	−0.010 *
Emergency care	−0.057 *	−0.055 *	−0.066 *	−0.045 *	−0.085 *	−0.057 *	−0.062 *	−0.042 *	−0.061 *	−0.035
Pharmaceuticals	−0.093 *	−0.011 *	−0.046 *	−0.007 *	−0.022 *	−0.003	0.001	0.004 *	−0.028 *	−0.002
Total HC Costs	−0.086 *	−0.030 *	−0.049 *	−0.017 *	−0.046 *	−0.019 *	−0.028 *	−0.011 *	−0.043 *	−0.016 *

Note: * *p* < 0.05. ^(a)^ Observed *CI*: concentration index of actual health care use. ^(b)^ *HI*: estimates of horizontal inequity in health care use (after controlling for need proxied by age, gender and number of comorbidities).

## Data Availability

The database is property of the Basque Health Service and access is restricted.
